# Biomass-Derived Flexible Carbon Architectures as Self-Supporting Electrodes for Energy Storage

**DOI:** 10.3390/molecules28176377

**Published:** 2023-08-31

**Authors:** Dehong Yang, Peng Xu, Chaofan Tian, Sen Li, Tao Xing, Zhi Li, Xuebin Wang, Pengcheng Dai

**Affiliations:** 1 College of New Energy, China University of Petroleum (East China), Qingdao 266580, China; 2 New Energy Division, National Engineering Research Center of Coal Gasification and Coal-Based Advanced Materials, Shandong Energy Group Co., Ltd., Jining 273500, China; 3 National Laboratory of Solid State Microstructures (NLSSM), Collaborative Innovation Center of Advanced Microstructures, College of Engineering and Applied Sciences, Nanjing University, Nanjing 210093, China; wangxb@nju.edu.cn

**Keywords:** biomass, carbon, flexible, self-standing electrodes, energy storage

## Abstract

With the swift advancement of the wearable electronic devices industry, the energy storage components of these devices must possess the capability to maintain stable mechanical and chemical properties after undergoing multiple bending or tensile deformations. This circumstance has expedited research efforts toward novel electrode materials for flexible energy storage devices. Nonetheless, among the numerous materials investigated to date, the incorporation of metal current collectors or insulative adhesives remains requisite, which entails additional costs, unnecessary weight, and high contact resistance. At present, biomass-derived flexible architectures stand out as a promising choice in electrochemical energy device applications. Flexible self-supporting properties impart a heightened mechanical performance, obviating the need for additional binders and lowering the contact resistance. Renewable, earth-abundant biomass endows these materials with cost-effectiveness, diversity, and modulable chemical properties. To fully exploit the application potential in biomass-derived flexible carbon architectures, understanding the latest advancements and the comprehensive foundation behind their synthesis assumes significance. This review delves into the comprehensive analysis of biomass feedstocks and methods employed in the synthesis of flexible self-supporting carbon electrodes. Subsequently, the advancements in their application in energy storage devices are elucidated. Finally, an outlook on the potential of flexible carbon architectures and the challenges they face is provided.

## 1. Introduction

At present, the burgeoning growth of wearable sensors, the portable electronics industry, and healthcare have engendered a noteworthy expansion of fundamental research and commercialization in the domain of flexible energy storage, alongside its supporting components [1,2]. To achieve superior performance in flexible devices, it is imperative that their energy storage components maintain efficiency, reliability, and integration, even when subjected to bending, stretching, twisting, or intricate deformations [3,4]. Metallic current collectors, such as nickel foam and stainless steel mesh, exhibit excellent mechanical properties and electrical conductivity, rendering them highly promising as flexible substrates for constructing energy storage devices [5]. Nonetheless, these materials are still hampered by their substantial weight and bulk density, imposing limitations on their widespread application [3]. Moreover, flexible electrodes founded on such substrates often necessitate the incorporation of insulating polymer binders, such as polytetrafluoroethylene and poly (vinylidene fluoride) [6]. The introduction of these insulating binders leads to additional costs, unwarranted weight, and enhanced contact resistance, further impeding their practical utilization [7]. Consequently, there arises an imperative need to fabricate electrically conductive free-standing active materials endowed with well-developed structures and exceptional mechanical properties, which is crucial to fostering the advancement of sustainable next-generation flexible energy storage devices.

Biomass, comprising cotton [8], silk [9], wood [10], and fibers [11], offers a diverse array of inherent advantages, including ample availability, sustainability, and biocompatibility [3]. The innate and superior chemical functionality endows biomass with substantial potential for synthesizing various morphological and functional architectures. Notably, employing specific methods, such as carbonization [12], electrostatic spinning [13], and vacuum filtration [14], facilitates the production of flexible carbon membranes/monolithic free-standing materials, which are distinguished by their large specific surface area, abundant functional groups, and three-dimensional (3D) mesh structure. Moreover, exceptional folding and bending capabilities, coupled with excellent electrical conductivity, render these materials highly promising for utilization as flexible electrode materials in energy storage devices, encompassing metal-ion batteries [15], supercapacitors [16], and metal–air batteries [17], thus ideally fulfilling the requirements of next-generation flexible electronic devices.

**Figure 1:**
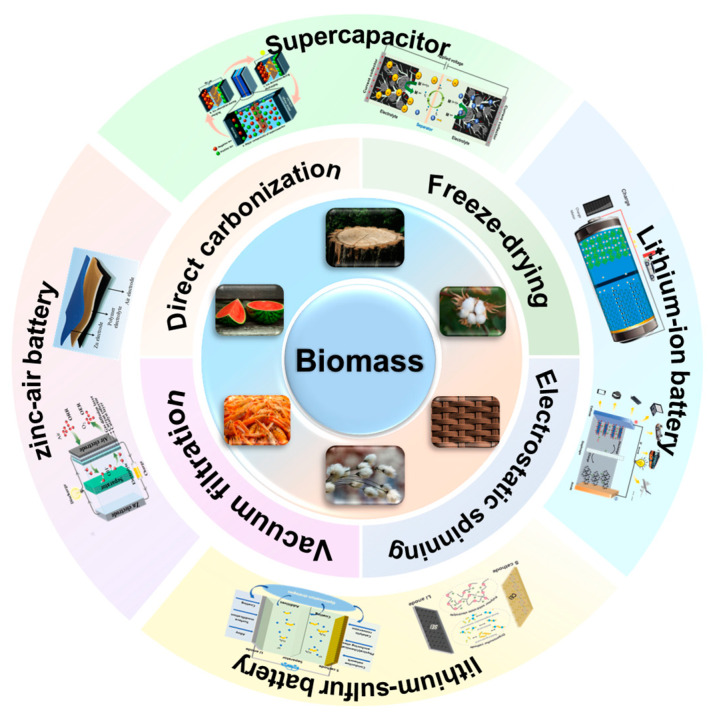
Schematic illustration depicting the main topics covered: biomass sources, synthesis methods, and the potential applications of flexible electrodes.

In view of the rapid development and broad prospects in the field of flexible energy storage devices, this review endeavors to establish a close association between biomass-derived carbon films/monoliths and flexible electrode materials, aiming to provide new ideas for designing and fabricating advanced energy storage systems. We delve into the vast potential of green and environmentally sustainable biomass as a source for multidimensional flexible freestanding electrodes with diverse properties. Considerable emphasis is placed on the abundant and sustainable biomass source, the uncomplicated and practical preparation method, and the highly promising application prospects in energy storage devices for the flexible electrodes (Figure 1). This review aims to offer theoretical and technical insights into the advancement of nanotechnology concerning self-supported carbon architectures derived from biomass and the expansion of their application areas as advanced functional materials.

## 2. Synthesis

### 2.1. Resources

#### 2.1.1. Cellulose

Cellulose, composed of linear chains interconnected by covalent β-1-4-glucosidic bonds formed from repeating β-D-glucopyranose units, stands as the most abundant biopolymer in the natural world [11]. The remarkable characteristics of eco-friendliness, renewability, and cost-effectiveness render it ubiquitous across diverse realms, including both daily life and industrial applications [18]. Significantly, cellulose can arrange into 3D porous structures, showcasing exceptional mechanical strength while retaining unparalleled flexibility. This intrinsic quality has led to remarkable opportunities for its utilization as a free-standing electrode material [18,19].

**Figure 2:**
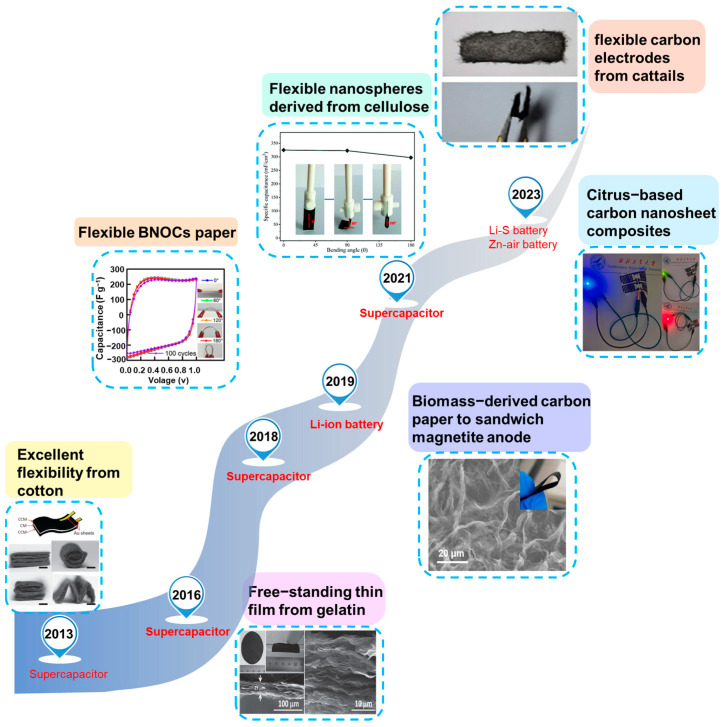
Development history of cellulose-based flexible electrode materials (from Refs. [8,22,23,24,25,26,27]).

Natural materials, such as cotton, flex, and leaves, consisting of over 90% entangled microscale cellulose fibers, exhibit sustainability, abundance, and inherent flexibility [20,21]. As early as 2013, Lou et al. proposed the direct carbonization of cotton for use as flexible electrodes in supercapacitors [8], and since then, we have witnessed the flourishing development of such materials in flexible devices (Figure 2) [22,23,24,25,26,27]. Recently, bendable and foldable cellulose-based materials have become suitable substrates for flexible energy storage devices, including supercapacitors [16], lithium-ion batteries [28], zinc–air batteries [29], and others [30].

Paper, being one of the oldest flexible materials, holds great promise as a precursor owing to its abundant availability, cost-effectiveness, lightweight, recyclability, and inherent bendability [31]. In particular, the unique web-like morphology lends cellulose paper well to serving as a candidate for the formation of carbon fiber-web materials [32].

**Figure 3:**
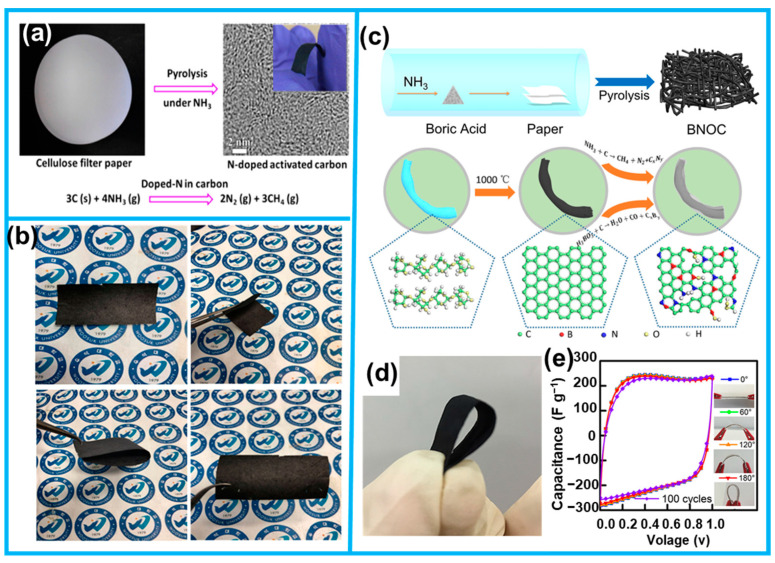
(**a**) Schematic presentation and digital photographs of the N-doped carbonized cellulose filter paper. (**b**) Optical images of as-prepared porous carbon fiber paper from Lokta paper. (**c**) Process method and schematic mechanism of synthesizing B, N, and O co-doped carbon paper. (**d**) Digital picture of BNOC-30. (**e**) The cyclic voltammetry curves when the capacitor is folded at different angles (from Refs. [23,33,34]).

Ji et al. achieved the successful prepared on N-doped flexible carbon materials by annealing filter paper as a precursor under ammonia (Figure 3a), which can be directly employed as self-supporting electrodes for supercapacitors [33]. Building upon this work, Hant et al. utilized KOH activation to modulate the pore structure and surface area of the carbonized paper, which not only improved the flexibility but also enhanced the electrochemical properties (Figure 3b) [34]. Moreover, our group successfully synthesized a 3D interconnected carbon microfiber network paper (BNOC) with controllable boron, nitrogen, and oxygen co-doping through chemical vapor-phase etching and heteroatom engineering process, employing filter paper as the substrate (Figure 3c,d). This unique network structure facilitated efficient electron transport due to its spatial continuity, while the layered porous structure offered a large specific surface area and rapid endoporous ion transport, rendering BNOC highly efficient in supercapacitors (Figure 3e) [23].

In addition to natural plant cellulose, bacterial cellulose (BC) serves as a notable precursor for flexible electrode materials, typically produced through microbial fermentation, showcasing a substantial aspect ratio (diameter: 20–100 nm; length: 1–10 μm) [35]. Its unique 3D interconnection network, rheological properties, and robust intra/inter-chain non-covalent interactions play a significant role in establishing a stable and sturdy structure [3]. This distinctive nano-fibrous architecture ensures BC’s exceptional mechanical durability, imparting reliability and resilience during bending, stretching, and torsion [36].

**Figure 4:**
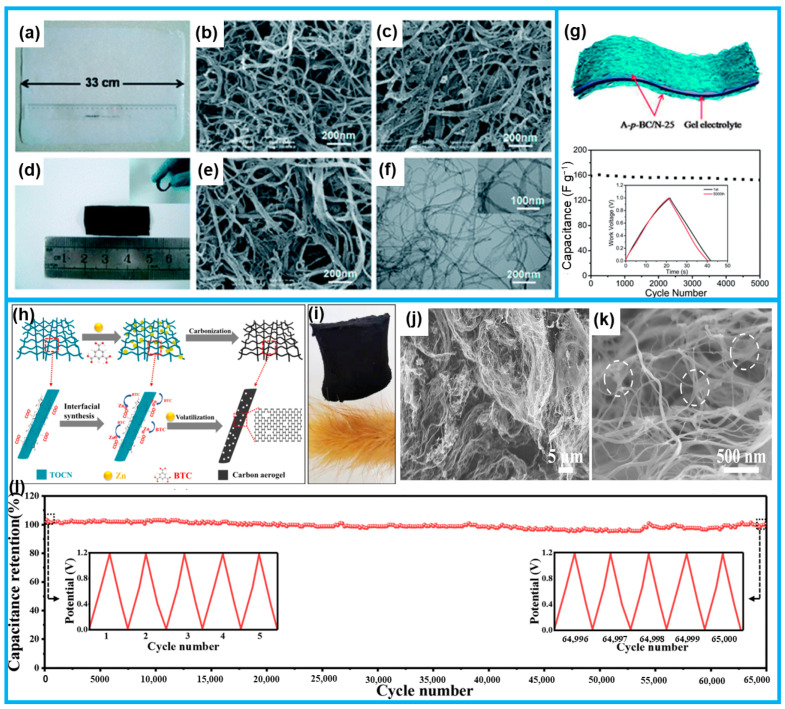
(**a**) Digital image of bacterial cellulose. (**b**,**c**) SEM images of the carbonized and activated BC. (**d**) Typical free-standing flexible BC-N being bent. (**e**,**f**) SEM and TEM images of BC-N. (**g**) The cycling stability of BC-N as a flexible electrode for supercapacitors. The 1st and 5000th cycles’ charge–discharge curves are shown inset. (**h**) Schematic of the synthesis and (**i**) photograph of the carbon aerogel. (**j**,**k**) SEM images of carbon aerogel. (**l**) Stability as a self-supporting electrode. (From Refs. [37,38]).

Yu et al. fabricated nitrogen-doped flexible carbon films (designated as BC-N) exhibiting a 3D interconnected network of closely spaced nanowires (10–20 nm) and interlinked voids, utilizing bacterial cellulose as the substrate (Figure 4a–f). The flexible supercapacitor, which was designed using BC-N as the basis, showed outstanding flexibility (Figure 4d) and demonstrated excellent stability with five thousand consecutive cycles (Figure 4g) [37]. Yang et al. synthesized hierarchically porous carbon aerogels with interconnected 3D nanofiber networks, which was accomplished by employing 2,2,6,6-tetramethylpiperidin-1-oxyl (TEMPO) oxidized bacterial cellulose as a precursor. The oxidized cellulose had anionic charges and demonstrated enhanced dispersibility in water. To further enhance the structure and porosity, the nanofibrils were thoughtfully supplemented with Zn-1,3,5-benzene tricarboxylic acid (Zn-BTC) (Figure 4h). The remarkable affinity between Zn^2+^ and bacterial cellulose played a pivotal role in the subsequent carbonization/evaporative etching process, giving rise to an abundance of defects and a well-defined distribution of micro- and mesopores within the nanofibers derived from bacterial cellulose, ultimately forming the anticipated hierarchical porous structure (Figure 4j,k). Notably, the resulting aerogel served as a bondless self-supporting electrode for supercapacitors, exhibiting remarkable stability (Figure 4i) [38]. By hybridization (e.g., carbon nanotubes [39,40,41], graphene [42], Mxene [43,44], and metal compounds [45]), polymerizations [46,47], and carbonization [48,49], bacterial cellulose can be transformed into a dependable and enduring flexible electrode material for energy storage devices.

#### 2.1.2. Lignin

Lignin assumes a crucial role as a structural material during the developmental process of plant support tissues, vascular plant support tissues [50]. Within natural wood, lignin establishes physical or chemical linkages with cellulose in the cell wall, enhancing wood hardness, thus forming the groundwork for the application of carbonized wood and even raw wood as flexible electrode materials [50,51]. For example, Jiao’s team employed the strong hydrogen-bonding interactions between the aniline molecule and the -OH groups of the raw wood matrix to facilitate the complete self-assembly of polyaniline nanorods. The self-standing integrated material composed of polyaniline/wood can be used as high-performance electrodes for supercapacitors [52]. Apart from the inherent properties of the raw wood itself, subjecting it to high-temperature carbonization transforms it from an insulator to a higher surface area, electrically enhanced conductor. Simultaneously, it retains its 3D interconnected network, pore properties, and derivatized functional groups, rendering it an excellent self-supporting and thick electrode material for energy storage applications [16,53,54].

**Figure 5:**
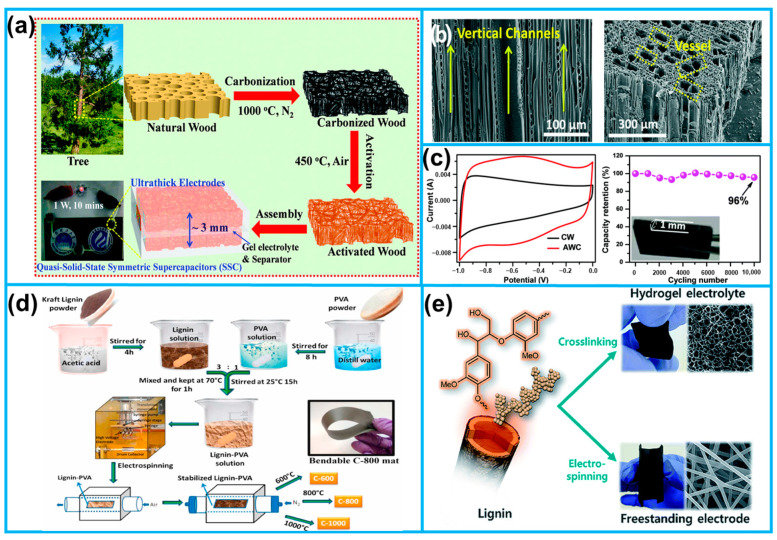
(**a**) The synthesis of activated wood carbon (AWC) monoliths. (**b**) Cross-sectional and top-view SEM images of the AWC. (**c**) CV curves at 1 mA cm^−2^ and cyclic stability of AWC; the inset is a digital photo of AWC. (**d**) Schematic diagram of carbon nanofiber film synthesized from sulfate lignin. (**e**) Schematic of the preparation of all-lignin-based flexible supercapacitors from lignin as a raw material (from Refs. [55,56,57]).

Liu developed a sustainable surface engineering methodology that effectively enhanced the energy density of supercapacitors with ultra-thick electrodes derived from renewable natural wood (Figure 5a) [55]. Benefiting from oxygen-containing groups, excellent 3D conductive networks, and layered micro/nanoporous structure with low curvature pathways (Figure 5b), the freestanding thick electrodes showed an unexpected high performance and demonstrated excellent cycling stability, retaining 96% of initial capacity even after undergoing 10,000 cycles (Figure 5c). Wu further functionalized the natural wood with the metal organic framework (ZIF-67). The carbonized ZIF-67 was highly distributed across the cell walls of the wood, culminating in a substantial enhancement of both graphitization and electrical conductivity within the carbon material [54].

In addition to natural wood, industrial lignin is primarily composed of alkali lignin [57,58] and kraft lignin [56,59], which are rich in aromatic groups and heteroatoms, and can be transformed into high-quality carbon architectures suitable for use as flexible electrode materials through specific methods and thermochemical conversion processes. Dhakate et al. employed renewable lignin sulfate as a precursor to thermally synthesize a carbon nanofiber film characterized by flexibility and foldability (Figure 5d). These exceptional properties position it as a promising candidate for a flexible self-supporting electrode in supercapacitors [56]. Ho Seok Park et al. utilized alkali lignin as the raw material to prepare lignin-based hydrogels as the electrolyte and carbonized lignin fibers as flexible electrodes (Figure 5e). The crosslinked network of the lignin hydrogel electrolyte exhibited high ion conductivity and mechanical integrity, while the independent flexible composite electrode demonstrated a remarkable charge storage capacity and kinetic performance through interconnected porous channels. Assembled together, the all-lignin-based flexible supercapacitor exhibited both flexibility and durability, maintaining high capacitance even at various bending angles, offering a novel strategy for developing new types of flexible supercapacitors [57].

#### 2.1.3. Silk

**Figure 6:**
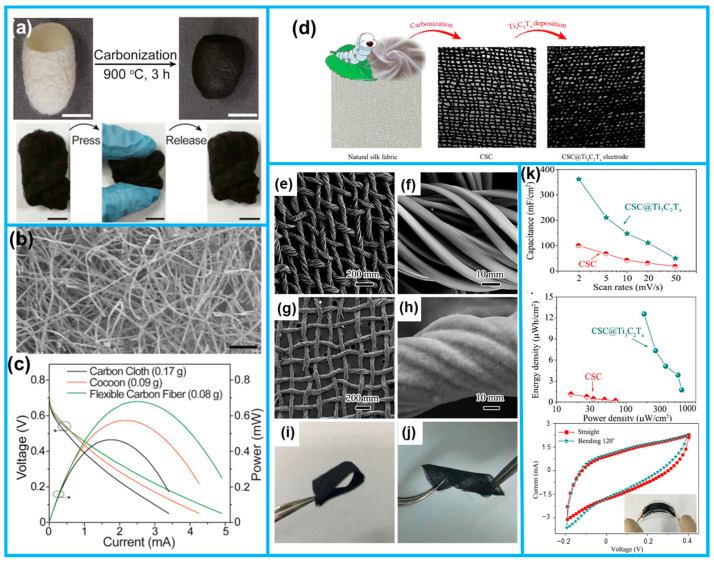
(**a**) Schematic presentation and digital photographs of the carbonized silk cocoon. (**b**) SEM images of flexible carbon materials obtained by the carbonization of silk cocoons. (**c**) Fuel cell performance curves with different anodes. (**d**) Schematic synthesis of CSC and CSC@Ti_3_C_2_T_x_. SEM images of (**e**,**f**) CSC and (**g**,**h**) CSC@Ti_3_C_2_T_x_. Digital pictures of (**i**) bent and (**j**) twisted CSC@Ti_3_C_2_T_x_. (**k**) Analysis and comparison of electrochemical properties of CSC and CSC@Ti_3_C_2_T_x_. (From Refs. [60,62]).

Silk predominantly comprises naturally occurring pliable protein fibers produced by a class of arthropods, affording distinct advantages, such as biocompatibility and biodegradability [9,60]. The structural flexibility of silk is ascribed to the formation of stable disulfide bonds, conferring exceptional tensile strength upon the material [4]. Furthermore, the surface of silk features a substantial array of functional groups, including amino, carboxyl, and hydroxyl groups, thereby presenting opportunities for functionalization [61]. Consequently, silk emerges as an optimal precursor for flexible electrode materials. Huang et al. successfully conducted the carbonization of silk, resulting in the production of nitrogen-rich pseudographitic anodes with adjustable flexibility (Figure 6a,b). It exhibited exceptional mechanical properties and, when utilized as portable anodes in fuel cells, achieved a maximum weight power density exceeding that of carbon cloth by more than 2.5 times (Figure 6c) [60].

Wang et al. achieved the direct carbonization of silk fabric, resulting in a conductive, flexible, and self-supporting substrate (CSC) [62]. Subsequently, they further modified this substrate using nano-thickness Ti_3_C_2_T_x_ flakes to create MXene-coated flexible fabric electrodes, referred to as CSC@Ti_3_C_2_T_x_ (Figure 6d). The SEM images demonstrated the uniform and robust integration of nano-thickness Ti_3_C_2_T_x_ flakes into the conductive fabric support (Figure 6e–h). Notably, the modified CSC@Ti_3_C_2_T_x_ exhibited remarkable flexibility, rendering it easily bendable and twistable (Figure 6i,j). The improved electrochemical properties and consistent behavior were observed during testing of the supercapacitors assembled with CSC@Ti_3_C_2_T_X_, where no significant changes in the CV curves were detected, even after subjecting the capacitors to numerous bending and twisting deformations (Figure 6k). This outcome strongly implies that silk-based flexible materials hold immense potential for widespread applications in energy storage devices. In addition, the unique biocompatibility of silk renders it a harmless substrate for incorporation in wearable devices designed for human health monitoring and motion-tracking applications [4,63].

### 2.2. Methods

#### 2.2.1. Direct Carbonation

As previously stated, specific natural and processed materials, including raw wood [10,54,64,65,66], cotton [8,67,68,69,70,71,72,73], flax [74], textiles [75,76,77,78,79,80,81,82,83,84], silk [60,62,85,86,87,88,89], and paper [12,23,24,33,34,90,91,92,93], inherently exhibit inherent flexibility, pliability, and foldability. These flexible precursors can be easily transformed into high-quality carbon architectures with superior electron conductivity and charge transfer advantages through a simple one-step high-temperature carbonization process. By manipulating the carbonization temperature, reaction atmosphere, and other pertinent parameters, the facile modulation of the pore structure and element doping can be achieved in the carbon architectures. Thus, direct carbonization is considered the simplest and most efficient method for synthesizing self-supporting electrode materials with outstanding electrical conductivity, mechanical flexibility, and macroscopic structure [94].

**Figure 7:**
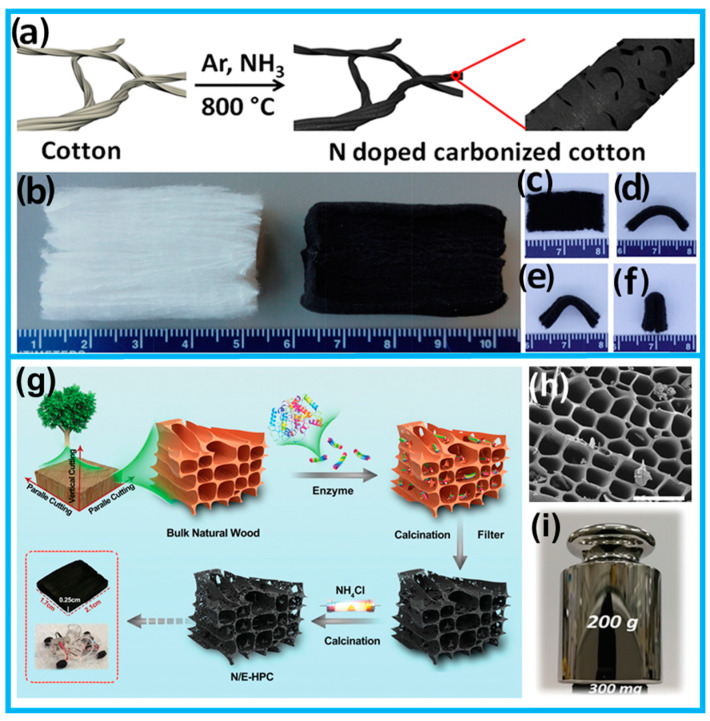
(**a**) Schematic diagram of nitrogen-doped cotton carbide synthesized by direct carbonization. (**b**) Digital and (**c**–**f**) SEM images of N-doped cotton carbide. (**g**) Schematic synthesis of nitrogen-doped porous carbon derived from enzymatically dissolved wood (N/E-HPC). (**h**) SEM image of N/E-HPC. (**i**) Image of N/E-HPC flat support 500 g weights (from Refs. [64,67]).

For instance, Tour employed direct pyrolysis under an ammonia atmosphere to obtain an impressively flexible mesoporous nitrogen-doped carbonized cotton from untreated cotton (Figure 7a–f), which exhibited a remarkable electrochemical performance when applied in supercapacitors [67]. Peng et al. demonstrated the direct carbonation of enzymatically hydrolyzed wood under ammonium chloride conditions to synthesize nitrogen-doped porous carbon (Figure 7g). The presence of a hierarchical pore structure (Figure 7h) and excellent mechanical strength (Figure 7i) made it a highly suitable non-metallic electrode for zinc–air batteries [64]. Wang et al. employed a highly effective electrode structure engineering approach to fabricate N-S co-doped carbon interfaces with excellent conductivity and substantial porosity on cellulose fabrics. The resulting pomegranate-like structure facilitated enhanced ion and electron transport and exhibited a significant integrated capacitance [82]. Indeed, in addition to the previously mentioned materials, certain natural plants, such as watermelon [95] and pomelo [96], inherently possess flexible and thickness characteristics, rendering them suitable for direct utilization as electrode materials following the carbonization process. However, carbon architectures obtained through direct carbonization methods primarily exhibit a dominance of micropores, with a limited mesoporous content. Consequently, pore engineering techniques are commonly employed to enrich the pore structure and further optimize their performance.

#### 2.2.2. Freeze Drying and Carbonation

Aerogels are types of gels composed of 3D nanostructured solid networks, featuring continuous nanoscale pores within an open 3D framework that facilitates the efficient diffusion/mass transfer of liquid/gaseous analytes or substrates [97,98]. Biomass materials, possessing naturally abundant porous structures, excellent hydrophilicity, and numerous active functional groups, can be readily transformed into aerogels using the facile freeze-drying process [38,99,100,101,102,103,104]. In the process of freeze-drying, water within the biomass material crystallizes into ice, and subsequently sublimates [104]. Throughout this progression, the ice crystals’ structure remains preserved within the resulting aerogel, thereby enhancing the porous architecture of the biomass material [105,106]. Thus, by employing the freeze-drying process, precise control over the aerogel’s pore structure is attainable through the modulation of ice crystal size, distribution, and morphology [107]. Subsequently, a networked structure can be reinforced through high-temperature carbonization, resulting in the formation of flexible carbon aerogel monoliths [108]. These 3D porous carbon monoliths with high specific surface areas exhibit enhanced mass transfer dynamics, improved charge storage, and electrolyte accessibility, which can be directly employed as adhesive-free electrodes, eliminating the need for insulating binders [109,110].

**Figure 8:**
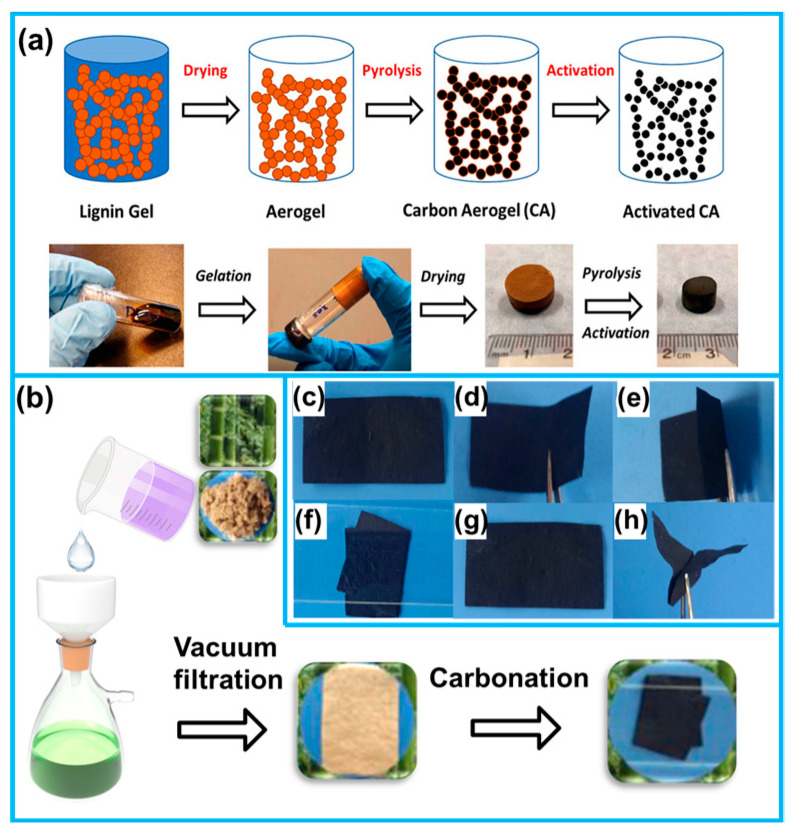
(**a**) Schematic synthesis of lignin-derived ultra-light carbon aerogels and corresponding optical photographs. (**b**) Fabrication of ultra-flexible carbon fiber membranes inspired by papermaking methods. (**c**–**h**) Digital photographs of ultra-flexible carbon nanofibers under different degrees of folding [59,121]).

Renneckar et al. harnessed industrially derived softwood kraft lignin to successfully synthesize carbon aerogels with an exceptional bulk density of 0.83 g/cm^3^ by gel carbonation. The advantageous characteristics, including a high specific surface area, well-suited hierarchical pore structure, ultra-lightweight, and remarkable flexibility, make it a promising, cost-effective candidate for supercapacitor applications (Figure 8a) [59]. Wang et al. achieved the successful derivation of cattail fiber aerogel by freeze-drying sodium chlorite-treated cattail fibers, followed by pyrolysis under a N_2_ atmosphere to obtain carbon aerogel. The resulting interconnected 3D network structure provided an exceptional platform for immobilizing pseudocapacitive active conducting polymer materials [111]. Notably, the utilization of templates during the preparation of gels is recognized as an effective method for designing structures, allowing control over parameters, such as pore size and porosity, which enhances the electrochemical performance of carbon-based materials [112,113].

To facilitate the integration of carbon architectures derived from biomass into energy storage devices, such as batteries or supercapacitors, it is necessary to mold the aerogels as well as the carbon-derived carbon aerogels. Vacuum filtration represents a continuous and straightforward physical separation technique used to isolate solids from liquids [114]. During the process, the liquid component is separated through permeation of the filter paper’s pores, while the desired functional material is deposited onto the filter paper’s surface, thereby forming a monolithic biomass membrane [114,115]. The thickness of the deposited film can be readily adjusted by controlling the concentration and filtration processes of the functional material mixture solution. Certain biomass particles, such as straw [116], carbon pellets [117], and others [25,26,118,119], can be naturally formed into films by the vacuum filtration method. Following carbonation, these films can be transformed into flexible carbon electrode materials suitable for diverse energy storage devices.

Xie et al. successfully fabricated monolithic nanocellulose membranes by the vacuum filtration–freeze drying technique. After undergoing carbonization, the resulting flexible carbon film exhibited a cross-linked structure characterized by numerous weld-like junctions, enabling it to function as an interconnected conductive network, thereby facilitating rapid electron transfer [120]. Inspired by papermaking technology, Xie et al. successfully prepared a hierarchical porous carbon fiber membrane from vacuum filtration and carbonization molding (Figure 8b). This ultra-flexible carbon fiber membrane exhibited excellent mechanical flexibility, allowing for repeated folding (Figure 8c–h). Moreover, it demonstrated outstanding electrochemical properties and could be directly employed as a freestanding electrode material for supercapacitors [121].

#### 2.2.3. Electrostatic Spinning and Carbonation

**Figure 9:**
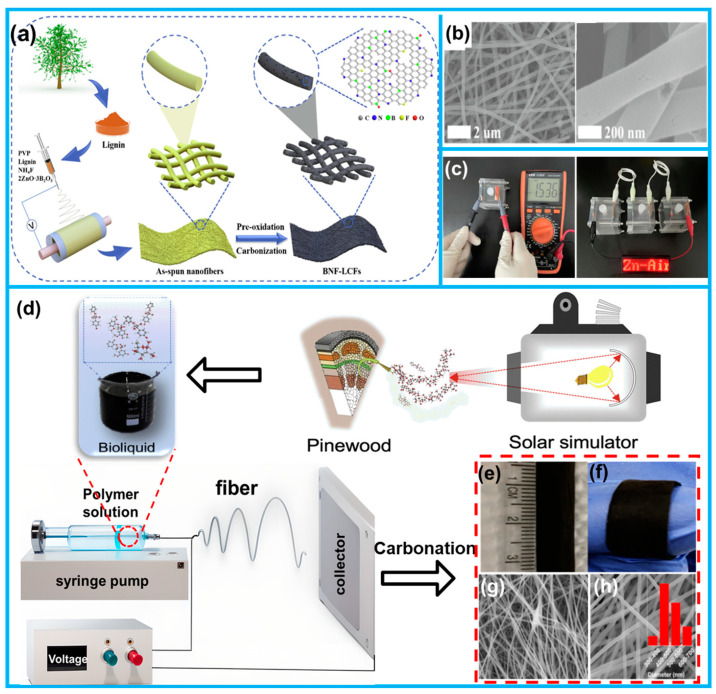
(**a**) Schematic of the synthesis method of BNF-LCFs. (**b**) SEM images of BNF-LCFs. (**c**) The open circuit voltage of liquid ZABs based on BNF-LCF and the performance of LEDs powered by three liquid ZABs assembled with BNF-LCFs. (**d**) Preparation process of pinewood as binder-free electrodes. (**e**,**f**) Photographs and (**g**,**h**) SEM images of flexible electrodes derived from pinewood. (From Refs. [126,128]).

Electrostatic spinning is a direct and continuous method for producing ultrafine fibers with diameters ranging from submicrons to a few nanometers [122], and the schematic is shown in Figure 9. This technique involves an electrohydrodynamic process, where droplets of a polymer solution or melt are electrically charged under a high electric field, giving rise to the formation of a liquid jet. The liquid jet subsequently undergoes stretching and elongation before being collected on a receiving device, resulting in a fiber mat that resembles a nonwoven-like fabric [123]. Through the electrostatic spinning method, the biomass feedstock can be transformed into flexible protofilaments and exhibit inherent and distinctive merits: (1) tunable fiber architecture: electrostatic spinning allows for the precise modulation of parameters, such as electric field intensity, spinning rate, and solution concentration, thereby enabling control over the fiber diameter, alignment, and structure, which proves exceptionally advantageous for the tailored design of flexible biomass-derived carbon materials [124]. (2) Manageable mechanical performance: the manipulation of fiber diameter and hierarchical arrangement empowers the regulation of the mechanical properties of the fiber mat. This characteristic allows materials to sustain both suppleness and robustness across diverse stress environments, thereby satisfying assorted practical requisites. (3) Porous and multifunctional attributes: ultrafine fibers produced via electrostatic spinning frequently manifest porous structures, contributing to a heightened specific surface area. Furthermore, the controlled incorporation of various biomass during the electrospinning process facilitates the attainment of material multifunctionality [125]. Importantly, the resultant products can maintain their original flexibility after carbonization, rendering them highly suitable for application as a flexible electrode material in energy storage devices [57,126,127,128,129,130,131,132,133].

Shi et al. synthesized lignin-based carbon nanofibers (BNF-LCFs) doped with a triple combination of boron, nitrogen, and fluorine using a process involving electrostatic spinning and carbonation [126]. The key components utilized in this process included sustainable lignin as the carbon source, polyvinylpyrrolidone as the spinning additive, zinc borate as the B source, and NH_4_F, which acted as both the fluorine source and a portion of the nitrogen source (Figure 9a). The incorporation of B, N, and F heteroatoms through triple doping resulted in the creation of abundant active sites, effectively modifying the electronic structure of the carbon material (Figure 9b). Notably, the BNF-LCF films exhibited excellent flexibility, making them highly adaptable for constructing solid zinc-air batteries (ZABs) that demonstrated outstanding mechanical adaptability and stability (Figure 9c). Fan harnessed solar energy to conduct the pyrolysis of pine wood, resulting in the production of a phenol-rich biofluid [128]. This biofluid served as an excellent precursor for the electrostatic spinning process (Figure 9d), enabling the synthesis of binder-free flexible electrode materials (Figure 9e–h). The resulting flexible nanopads exhibited exceptional reversibility and cyclic stability, showcasing their promising potential for use in energy storage devices. This innovative approach offers new perspectives for obtaining and utilizing solar biofluids for applications in the field of energy storage.

## 3. Application of Flexible Free-Standing Carbon Architectures from Biomass

Versatile carbon free-standing architectures derived from biomass exhibit a combination of advantageous characteristics, such as cost-effectiveness, exceptional thermal stability, large specific surface area, customizable surface functionality, and outstanding mechanical properties, rendering them highly promising in advancing sustainable development in the realm of green energy [134]. In this section, we demonstrate the applications of flexible free-standing carbon architectures in the domain of electrochemical energy storage and conversion, with a particular focus on three key aspects: supercapacitors, Li-ion batteries, and zinc–air batteries. The objective is to underscore the manifold functionality of flexible carbon architectures in the field of electrochemistry.

### 3.1. Supercapacitors

**Figure 10:**
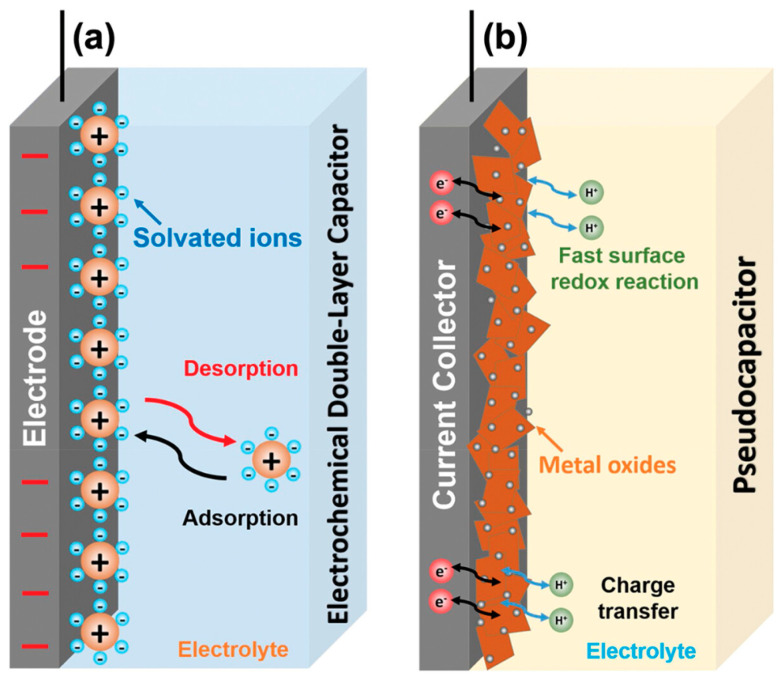
The charging illustrations of the (**a**) EDLC and (**b**) PC [136].

Supercapacitors, recognized as promising electrochemical energy storage devices, offer a remarkable power density surpassing that of conventional capacitors and have garnered substantial attention in recent research [135]. The supercapacitors are categorized based on charge storage mechanisms into three distinct types: the electrochemical double-layer capacitor (EDLC), pseudo-capacitor (PC), and hybrid supercapacitor (HCS) [136]. The EDLC operates by exploiting the phenomenon of electrostatic physical adsorption, rapidly and reversibly capturing anions and cations from soluble/organic electrolytes onto the positive and negative electrodes, forming a double-layer capacitor for charge storage (Figure 10a). On the other hand, pseudo-capacitors store energy through swift and reversible redox reactions taking place between the electrode surface and the electrolyte (Figure 10b) [137]. Generally, an EDLC demonstrates excellent power density and cycle stability among various types of supercapacitors. However, due to its charge storage mechanism relying on electrostatic adsorption, its specific capacitance remains relatively low. In contrast, PC electrodes, involving both capacitive Faradaic reactions and additional storage mechanisms entailing ion insertion, demonstrate an enhanced storage capacity and rate performance. The hybrid supercapacitor (HSC) represents a combination of the EDLC and PC, effectively integrating the charge storage mechanisms of double-layer capacitors and pseudo-capacitors, thus constituting a high-efficiency capacitor that embodies the advantages of both EDLC and PC [138]. In a hybrid supercapacitor, one half operates as an EDLC, while the other half functions as a PC, enabling the concurrent realization of the high specific power of the EDLC and the exceptional specific energy of the PC.

#### 3.1.1. Electric Double-Layer Capacitor (EDLC)

Considering that the EDLC relies on non-hydrodynamic charge accumulation at the electrolyte-electrode interface, the porous nature of electrode materials plays a crucial role in facilitating the penetration of the electrolyte into the electrode pores, thereby enhancing charge accumulation. Utilizing porous materials with a large surface area as electrodes can significantly improve the electrochemical performance of supercapacitors [139,140]. Graphene, carbon nanotubes (CNTs), and activated carbon are common carbon-based materials employed as EDLC electrode materials due to their substantial surface areas. However, these carbon-based materials often require the introduction of insulating binders, leading to unnecessary costs and restricting their applications in supercapacitors. An alternative approach involves converting biomass materials into flexible freestanding carbon architectures with low cost, excellent flexibility, high electrical conductivity [141,142,143]. These materials possess desirable porosity, mechanical strength, and an interconnected network structure that promote efficient charge transfer, holding promising potential for their utilization as standalone electrode materials or substrate materials in EDLC applications [144].

**Figure 11:**
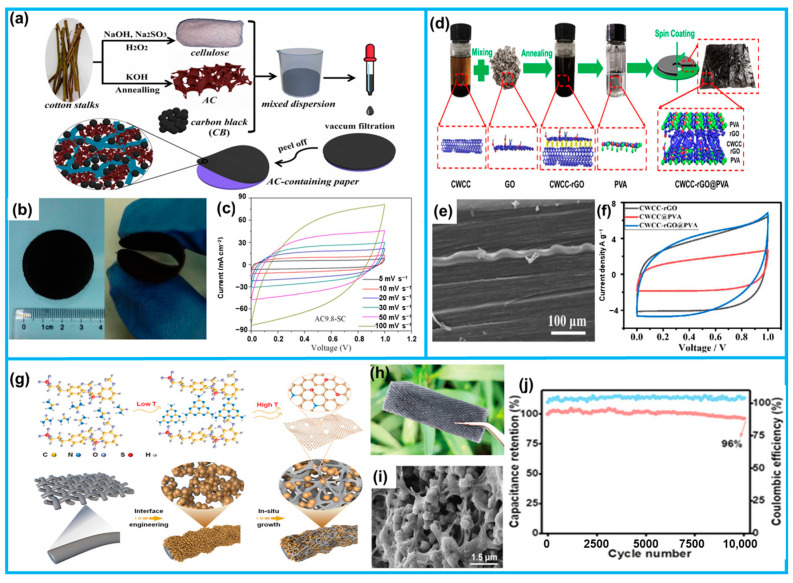
(**a**) Synthesis illustration process of AC from cotton stalks. (**b**) Photographic images of AC-containing paper. (**c**) Electrochemical properties of AC paper. (**d**) The preparation process of CWCC-rGO@PVA. (**e**) SEM picture of the CWCC-rGO@PVA hybrid. (**f**) CV curves of different composite materials. (**g**) Schematic illustration of the process of NS-(DA)n-cell. Optical photos (**h**) and SEM images (**i**) of the prepared NS co-doped carbon material. (**j**) Cyclic stability of the prepared electrode material (from Refs. [82,116,146]).

Wong employed cotton stalks as biomass precursors to successfully synthesize the flexible thin-film paper of carbonized activated carbon using a vacuum filtration method (Figure 11a,b) [116]. The flexible supercapacitors assembled using it as a flexible electrode exhibited excellent energy and power density values (0.89 mWh cm^−3^ and 209 mW cm^−3^, respectively, Figure 11c), along with outstanding cyclic reliability (97.1% capacitance retention after 10,000 cycles). Notably, this material can be utilized as a flexible substrate for the further fabrication of Fe_2_O_3_-decorated flexible electrodes. The assembled asymmetric supercapacitor exhibited an excellent electrochemical performance. In addition, biomass-derived carbon architectures can be used directly as electrode materials for supercapacitors apart from flexible substrates. Wood with unique cellular cavities and a porous structure, after high-temperature carbonization to obtain a uniform natural micron-sized tubular structure, is attracting considerable attention. Tang et al. successfully synthesized N and S co-doped carbon monolithic materials derived from raw wood (TARC-N) [145]. The porous structure and surface functionality were simultaneously optimized by heteroatom doping and a subsequent NH_3_ activation process, resulting in abundant active sites and an enlarged SSA (1438 m^2^ g^−1^). When used as a free-standing electrode, the resulting TARC-N exhibited a satisfactory specific capacitance of 704 F g^−1^ at 0.2 A g^−1^, and exhibited good stability over 5000 cycles.

In order to cope with the still poor mechanical strength of the electrodes derived directly from raw wood and to further improve the electrical performance, Ni designed a composite material called CWCC-rGO@PVA (Figure 11d) [146]. The innovation lies in the combination of the carbonized wood cell chamber (CWCC) as a substrate, graphene oxide (GO) in the form of C-C bonds, and polyvinyl alcohol (PVA) in the form of hydrogen bonds, resulting in a hybrid material with an outstanding electrochemical performance (Figure 11f). Additionally, the presence of PVA further enhanced the flexibility, deformability, and sensitive sensing properties, making the composite a suitable sensor to detect human movement. In order to achieve further improvements in the electrochemical performance, morphology modulation has emerged as an effective strategy for tailoring electrode materials. Wang et al. successfully synthesized lignosulfonate-derived N/S co-doped graphene-like carbon within interface-engineered cellulose textiles, utilizing the sacrificial template method (Figure 11g) [82]. The resulting pomegranate-like structure exhibited continuous conductive pathways and porous properties, which enabled sufficient ion/electron transport throughout the structure (Figure 11h,i). Consequently, the resulting flexible electrodes exhibited a substantial integrated capacitance of 335.1 F g^−1^ and demonstrated exceptional stability (Figure 11j), even when subjected to industrially applicable mass loading conditions of 19.5 mg cm^−2^, making them highly suitable for industrial applications. Furthermore, the utilization of the same electrode structure to fabricate a symmetric supercapacitor resulted in a notably heightened area capacitance of 3625 mF cm^−2^ and an impressive maximum energy density of 1.06 mWh cm^−2^, thereby highlighting the remarkable performance and substantial potential of this technology.

#### 3.1.2. Pseudo-Capacitor (PC)

The pseudo-capacitor (PC), known as a Faraday capacitor, is distinguished by its ability to store energy through rapid surface redox reactions, resulting in its high capacitance and exceptional rate capability. However, traditional electrode materials for PCs, such as transition metal oxides and conductive polymers, are plagued by inherent limitations, such as poor stability and inadequate conductivity [147]. These electrode structures often employ brittle materials, leading to restricted cyclic stability and diminished mechanical strength under stress. Conversely, biomass-derived self-supporting carbon materials exhibit a unique winding structure, exceptional conductivity, and flexibility, rendering them highly suitable as functional scaffolds in constructing the electrode body. Moreover, the surfaces of these materials are enriched with functional groups, which not only enable further functionalization but also facilitate ion transport, consequently contributing to the attainment of an excellent cycling performance for the electrode.

**Figure 12:**
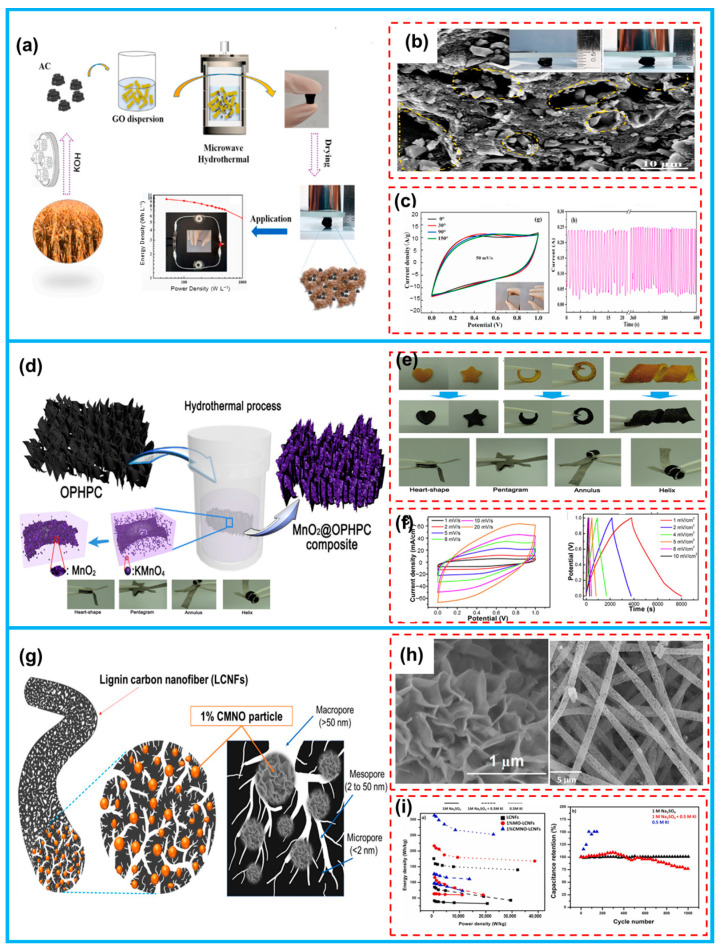
(**a**) The synthesis of AC3/G. (**b**) SEM and digital images of AC3/G. (**c**) The relative electrochemical properties of AC3/G. (**d**) The schematic illustration of fabrication of MnO_2_@OPHPC. (**e**) Photographic images of different shapes of orange peels. (**f**) CV and GCD curves of the flexible device. (**g**) Schematic representation of the synthesis of Ni-Co@MnO_2_-modified LCNFs. (**h**) SEM surface images of as-prepared LCNF-based materials. (**i**) Electrochemical energy storage performance of 1%CMNO-LCNFs. (From Refs. [131,148,149]).

Biomass-derived carbon-based materials possess abundant functional groups and surface modifications, as well as high large and porous structures, enabling them to exhibit pseudo-capacitive behavior. Consequently, they can be utilized as electrodes for PCs. Yu et al. achieved a successful combination of biomass-based carbon (AC) with graphene oxide through microwave-assisted treatment and freeze-drying, using straw-derived carbon as the biomass feedstock (Figure 12a). This innovative approach resulted in the development of high-density carbon composites (designated AC3/G) with a layered porous structure (Figure 12b), boasting densities as high as 1.23 g cm^−3^. Subsequently, when the AC3/G was used as a freestanding electrode to form a flexible supercapacitor, an impressive capacitance of 326 F cm^−3^ was attained at 0.5 A g^−1^. Moreover, the device demonstrated remarkable stability, showcasing the exceptional energy storage capabilities of this novel composite system (Figure 12c) [148].

Additionally, biomass-derived carbon with a distinctive 3D framework and exceptional electrical conductivity serves as a promising carrier for metal oxides with commendable electrochemical reactivity and high specific capacity. By amalgamating the two components, we can effectively harness their synergistic benefits, thereby mitigating challenges, such as capacity fade and structural degradation, ultimately leading to an extended operational lifespan of the capacitor. Sun et al. conducted a one-step carbonization process of orange peels to fabricate integral porous carbon (OPHPC, Figure 12d) [149], which exhibited a large specific surface area of 860 m^2^ g^−1^, natural porous channels, along with natural nitrogen doping. Utilizing OPHPC’s unique structure as well as its flexibility (Figure 12e), they synthesized a composite electrode (MnO_2_@OPHPC) through a simple hydrothermal process. The resulting composite electrode demonstrated both homogeneity and a high loading of MnO_2_, and exhibited a remarkable capacitance value of 3987 mF cm^−2^ (Figure 12f).

Varol Intasanta and colleagues employed an innovative hybrid Faradaic architecture to enhance the performance of carbon-based supercapacitors. The emphasis was placed on adopting a simple, cost-effective, and environmentally conscious method to synthesize lignin carbon nanofibers (LCNFs), which were subsequently combined with flower-like and intrinsically capacitive nanoparticles (Ni-Co@MnO_2_). By meticulously designing the hybrid composition, nanostructures, and active redox reactions, the researchers successfully boosted the specific capacitance from 129 F g^−1^ to an impressive 303 F g^−1^ across various electrolytes. Remarkably, in the favorable mixed electrolyte (1 M Na_2_SO_4_ + 0.5 M KI), the specific capacitance reached an exceptional 400 F g^−1^, and the stability persisted even after subjecting the supercapacitor to 1000 GDC cycles [131].

#### 3.1.3. Hybrid Supercapacitor (HSC)

The integration of charge storage mechanisms from double-layer capacitors and pseudo-capacitors has given rise to a class of high-performance energy storage devices termed hybrid supercapacitors (HSCs) [138]. Owing to the dual-charge storage mechanisms, HSCs exhibit an enhanced electrochemical performance compared to conventional EDLCs and PCs, thereby poised to fulfill elevated specific energy requisites. Electrode materials used for HSCs have diverse properties, including functionalized carbon [150], metallic hydroxides [151], and metal-organic frameworks (MOFs) [152], among other options [153]. Significantly, biomass-derived flexible carbon materials are highly regarded as promising candidates for HSC electrodes. This preference is attributed to their flexible well-interconnected porous frameworks, which eliminate the need for conductive additives or binders and display reduced internal resistance, thereby facilitating ion diffusion rates [154].

For instance, Zhang achieved a flexible self-supported 3D MXene/biomass/chitosan aerogel (MBC) carbon aerogel by the internal incorporation of 2D MXene nanosheets and external encapsulation with chitosan “cages” within radish slices [154]. The orderly arrangement of the well-defined structure effectively prevented the self-stacking of the MXene layers, while the macroscopic 3D aerogel architecture promoted the rapid diffusion and transfer of electrolyte ions. Through freeze-drying and annealing, a 6% MBC-doped pseudo-capacitive electrode (MBC-6) exhibited a volumetric capacitance of 1801.4 mF/cm^3^ at a scan rate of 2 mV/s. Furthermore, assembled binder-free asymmetric supercapacitors demonstrated an exceptionally high volumetric energy density of 33.4 Wh/L, an 82% capacitance retention rate, and an extended cycling life of 50,000 cycles at a high current density of 10 mA/cm^3^. Huang et al. devised a mechanically robust and electrochemically superior flexible HSC electrode membrane by employing low-cost, renewable kelp and bacterial cellulose as carbon precursors and flexible substrates, respectively [155]. The rational design of the surface area and pore volume facilitated efficient contact between the electrolyte ions and active sites, thereby expediting the ion diffusion rates and electron transfer kinetics. Simultaneously, the distinctive structure of Co_3_O_4_ nanosheets provided abundant active sites, contributing to additional pseudo-capacitance. This flexible HSC operated within a wide voltage window of 1.6 V and delivered high energy density (0.39 mWh cm^−2^) and power density (21.7 mW cm^−2^). Furthermore, the carbon-based material enhanced the cyclic stability of the electrode material, resulting in a remarkable capacitance retention rate of 94.5% after 10,000 cycles.

As previously explained, flexible carbon architectures derived from biomass demonstrate unparalleled merits, positioning them as the most promising electrode materials for EDLCs, PCs, and HSCs. The remarkable flexibility and inherent self-support of these carbon electrodes enhance their mechanical performance, thereby reducing the reliance on insulating binders, resulting in diminished contact resistance and lowered manufacturing costs. The distinctive 3D conductive network architecture facilitates superior pathways for ion and electron transport, thereby enhancing the electrochemical performance. The extensive availability of biomass sources and versatile design methods empower the tailoring of pore structures, shapes, and properties of biomass-derived flexible carbon materials to match specific requirements. Furthermore, the naturally occurring heteroatoms within the biomass not only enable the in situ doping of carbon materials but also serve as anchoring sites for metals, yielding high-activity catalytic centers to enhance the electrochemical performance. However, biomass-derived flexible carbon architectures still need to overcome certain limitations, such as conductivity challenges, heterogeneity resulting from diverse growth conditions, and constraints in specific application contexts, to fully harness their potential as electrode materials.

### 3.2. Lithium Batteries

#### 3.2.1. Lithium-Ion Battery (LIB)

Since 1991, lithium-ion batteries (LIBs) have undergone remarkable advancements and have established themselves as the most mature battery technology in the New Energy market with high voltage, energy density, and cycling stability properties [156]. However, continuous progress is required in the field of novel electrode materials and innovative structural designs to further enhance lithium storage performance in LIBs [157]. Biomass-derived flexible free-standing carbon architectures exhibit excellent mechanical properties and flexibility, which can supply conductive networks for Li^+^ redeposition and reduce internal pressure with interfacial fluctuations, and are considered to be highly promising and cost-effective solutions for the production of superior carbon-based electrodes.

**Figure 13:**
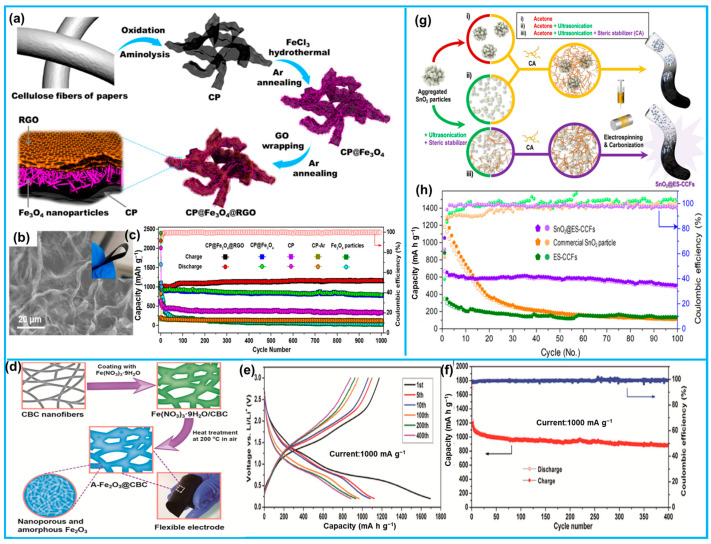
(**a**) Schematic synthesis of CP and CP@Fe_3_O_4_@RGO composites. (**b**) SEM and digital images of CP@Fe_3_O_4_@RGO. (**c**) Stability of lithium-ion batteries assembled with different electrode materials. (**d**) The synthetic diagram of the A-Fe_2_O_3_@CBC composite. (**e**,**f**) The typical charge/discharge profiles and long-term cycle curves of A-Fe_2_O_3_@CBC. (**g**) Synthesis schematic of highly dispersed SnO_2_ encapsulated in carbon nanofibers by electrostatic spinning method. (**h**) Cycling stability of SnO_2_@ES-CCFs versus other electrode materials (from Refs. [24,101,129]).

When Li-ion batteries function, repeated expansion–contraction leads to cracks on the new surface from chemical negative potential reactions with solvents and lithium, reducing the Coulombic efficiency (CE); hence, the greater the volume change, the worse the long-cycle cycling stability will be [158]. The loading of metal nanoparticles on 3D conductive net carriers and the encapsulation/intercalation method can enhance the CE and accommodate the volume change, enhancing the electron transport rate, respectively. Biomass-derived flexible carbon architectures can be considered as ideal 3D net carriers owing to the advantages of good conductivity, low cost, and volume variability [159]. Our team used cellulose as a precursor and O_2_-NH_3_-activated pyrolysis to obtain porous flexible carbon paper (CP) with a high specific surface area and electrical conductivity (Figure 13a,b) [24]. The carbon paper was further processed into CP@Fe_3_O_4_@RGO doped with Fe_3_O_4_ and reduced graphene oxide (GO) by further processing, which had a stable cycling performance with no additives and variable volume; an ultra-long cycling life of more than 2000 cycles can be realized at a high-capacity level of 1160 mAh g^−1^ (Figure 13c).

In addition, biomass-derived carbon architectures not only offer exceptional mechanical strength, but also a wealth of functional groups and interconnected 3D networks, which render them highly advantageous as substrates for anchoring active materials in energy storage devices. Sun et al. utilized a simple direct carbonization method to extract 3D carbonaceous aerogels from bacterial cellulose [101]. The aerogel was employed as a flexible platform, allowing the amorphous iron oxides to be tightly encapsulated on the carbonized bacterial cellulose nanofibers (Figure 13d). Due to the excellent compatibility between the highly dispersed amorphous Fe_2_O_3_ and the hierarchical pores of the carbon aerogel, the Fe_2_O_3_@CBC assembled as an electrode in half-cells exhibited an outstanding cycling stability and higher specific capacity (Figure 13e,f). Dong-Wan Kim et al. utilized cellulose acetate precursors to fabricate cellulose-derived carbon fiber/manganese dioxide composites by electrospinning [129]. Due to the strong interactions with cellulose-derived carbon fibers, SnO_2_ with a large quantity (46.4 wt%) was highly dispersed in the fibrous matrix, enabling the significant suppression of SnO_2_ degradation. As a result, the reversible capacity of this composite electrode reached up to 667 mA h g^−1^, and even after 100 cycles at a current of 200 mA g^−1^, it retained 76% of its stable capacity, which indicated that the stability far surpassed that of commercial SnO_2_ nanoparticles.

#### 3.2.2. Lithium–Sulfur Battery (LSB)

Lithium–sulfur batteries (LSBs) are an emerging storage energy technology, anticipated to become a promising choice for next-generation energy storage devices due to their significantly higher theoretical specific capacity in sulfur cathodes and superior energy density compared to current LIBs [160,161]. However, the practical implementation of LSBs still presents two significant challenges: (1) the low power utilization of sulfur and the limited conductivity of the Li_2_S/Li_2_S_2_ lithium product hinder efficient electron transfer from the sulfur cathode throughout the cycle, which leads to insufficient sulfur consumption and results in severe polarization [162,163,164]. (2) The polysulfides possess high solubility in the electrolyte, causing them to disperse within the battery, which gives rise to adverse reactions between the polysulfides and the lithium metal anode, significantly contributing to rapid decay and reduced coulombic efficiency in LIBs [165,166,167]. The significant role of porous carbon-based materials lies in their capability to accommodate dissociated sulfur, effectively managing volume changes occurring during electrochemical reactions, and restricting the shuttle loss of polysulfides at a physical level during cycling [168,169,170]. However, the utilization of some carbon architectures in LSBs is limited due to their high cost and complex preparation processes. In recent years, flexible carbon architectures derived from biomass, such as cotton and fruit peels, have emerged as excellent alternatives as carbon sources and have been recognized as the most promising electrode materials for lithium–sulfur batteries.

**Figure 14:**
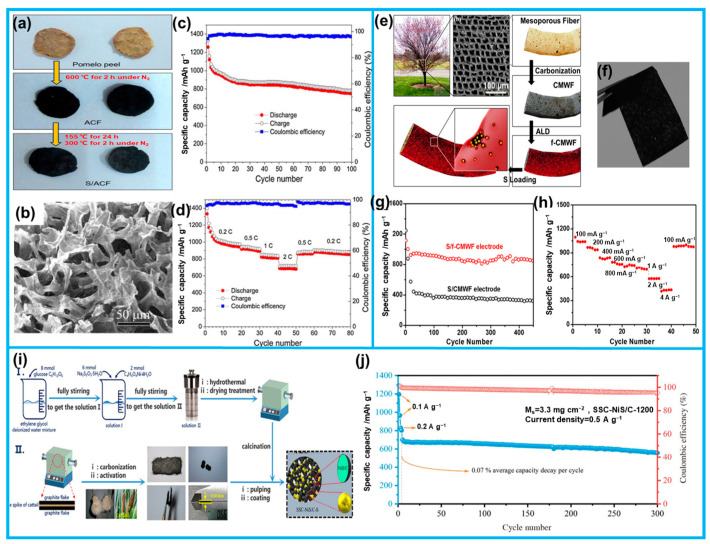
(**a**) Preparation process and (**b**) SEM image of S/ACF. (**c**,**d**) The cycling performances and muti-rate capability of the S/ACF electrode. (**e**) Schematic diagram of the process of synthesizing CMWF and f-CMWF. (**f**) The optical image of CMWF (**g**,**h**) The cycling stability and muti-rate capability of as-prepared electrode. (**i**) Illustration depicting the fabrication process of the SS-NiS/C cathode. (**j**) Long cycle performance at 0.5 A g^−1^ of SSC-NiS/C-1200 (from Refs. [27,96,171]).

Carbon architectures derived from biomass sources possess tunable structures and properties, enabling them to facilitate electron migration and serve as reservoirs for adsorbing conductive-limited and soluble polysulfides. These advantages make them an effective strategy for mitigating capacity degradation during the cycling process of lithium–sulfur batteries. Du et al. demonstrated the preparation of activated carbon with a microporous structure by the KOH activation of pomelo peels, and subsequently, they utilized solution permeation to incorporate sulfur into the microporous carbon framework, resulting in the formation of a composite porous carbon material (S/ACF, Figure 14a) [96]. The S/ACF material exhibited an initial discharge capacity of 1258 mAh g^−1^ at a rate of 0.2 C, while maintaining a high specific capacity of 750 mAh g^−1^ and 96% coulombic efficiency after 100 charge/discharge cycles (Figure 14c,d). Additionally, the S/ACF material effectively served as a storage layer, facilitating the adsorption of polysulfides.

Combining flexible carbon substrates with metal compounds is an effective strategy for stabilizing lithium–sulfur batteries. This composite material serves as a highly efficient sulfur-loaded interceptor, effectively adsorbing and confining polysulfides to reduce their dissolution and minimize their drift in the electrolyte, consequently alleviating polarization phenomena. These advancements provide promising solutions to enhance the performance and feasibility of LSB technology. Hu et al. utilized carbonized mesoporous wood fibers (CMWFs) as a substrate to accommodate sulfur (Figure 14e) [171]. The carbonized wood fibers retained their natural layered and mesoporous structure, enabling substantial sulfur loading (76 wt%) while effectively inhibiting the formation and dissolution of polysulfides in LSBs, thereby mitigating polarization phenomena. Subsequently, a 5 nm-thick Al_2_O_3_ film was fabricated on the CMWF using atomic layer deposition (ALD) technology, further tuning its pore size and significantly enhancing the stability of the sulfur cathode. As a result, the initial capacity reached 1115 mAh g^−1^, and even after 450 cycles, a reversible capacity of 859 mAh g^−1^ was maintained with excellent stability (Figure 14g,h). Zhang et al. presented an approach utilizing a biomass-derived, flexible self-supporting carbon (SSC) combined with NiS/C as a host for efficient sulfur storage in LSBs (Figure 14i) [27]. The prepared composite exhibited a remarkably high sulfur content of 5.3 mg cm^−2^, effectively achieving a dual physical/chemical blocking effect on polysulfides within the SSC-NiS/C electrode. The 3D SSC with its interwoven structure provided a large surface area and wide internal space, facilitating the accommodation of sulfur. Furthermore, the synergistic effect of SSC and NiS imparted strong physical immobilization and chemical anchoring capabilities to the polysulfides, effectively mitigating the shuttle effect. In the performance evaluation, the SSC-NiS/C-1200 electrode demonstrated excellent durability, retaining a residual capacity of 555.15 mA h g^−1^ after 300 cycles at a current density of 0.5 A g^−1^ under high-sulfur-loading conditions of 3.3 mg cm^−2^ (Figure 14j).

### 3.3. Zinc–Air Battery (ZAB)

Zinc–air batteries (ZABs), classified as a form of metal–air batteries, harnesses the chemical reaction between zinc metal and atmospheric oxygen to generate electric power [113]. In comparison to conventional alkaline batteries, ZABs exhibit a notably heightened energy density, thereby enabling the storage of a greater quantum of energy within a comparatively diminutive volume and mass [172]. Moreover, zinc, characterized by its widespread availability and relative affordability, provides an economically viable alternative when compared with metals, such as lithium, which are marked by constrained market supply and elevated costs. Consequently, the cost-effectiveness of manufacturing ZABs generally outpaces that of LIBs. Furthermore, when coupled with attributes, such as heightened safety and environmental compatibility, the robust trajectory of ZABs within the expansive market is substantiated, thus making them expected to become the next generation of energy storage devices [173].

The integral performance of ZABs mainly depends on the electrode kinetics of the oxygen reduction reaction (ORR) and oxygen evolution reaction (OER) in the discharge during discharge and charge processes [174]. Carbon-based architectures, due to their exceptional surface area, adjustable microstructure, and stable physicochemical properties, have emerged as widely adopted materials to optimize electrocatalytic reactions. However, conventional carbon nanotubes [175], graphene [176], and other carbon-based materials [177] encounter several drawbacks, including complex preparation procedures, high energy consumption, limited mechanical strength, and unsatisfactory yields. Biomass-derived flexible carbon architectures have emerged as a highly suitable candidate for carbonaceous components due to their cost-effectiveness, ready availability, and impressive mechanical properties [178]. These materials inherit porous structures, which contribute significantly to the efficient diffusion and transfer of electrolyte ions and oxygen molecules, owing to their large surface area. Moreover, the attributes of flexibility and free-standing structures reduce the necessity for non-conductive auxiliary bonding materials. This streamlined approach significantly simplifies the design and manufacturing processes of flexible air electrodes, enhancing electrochemical performance and circumventing the inherent limitations of traditional air electrodes, such as instability, rigidity, and unwieldy dimensions. As a result, the realization of flexible ZABs is substantially expedited. In addition, the carbon derived from the partial pyrolysis of carbohydrates contained oxygen-containing functional groups, enabling the adsorption and anchoring of transition metal ions through coordination. By harnessing and optimizing the intrinsic structural characteristics of biomass-based carbon and incorporating high-activity sites, the development of highly efficient electrocatalysts for ZABs is envisioned.

**Figure 15:**
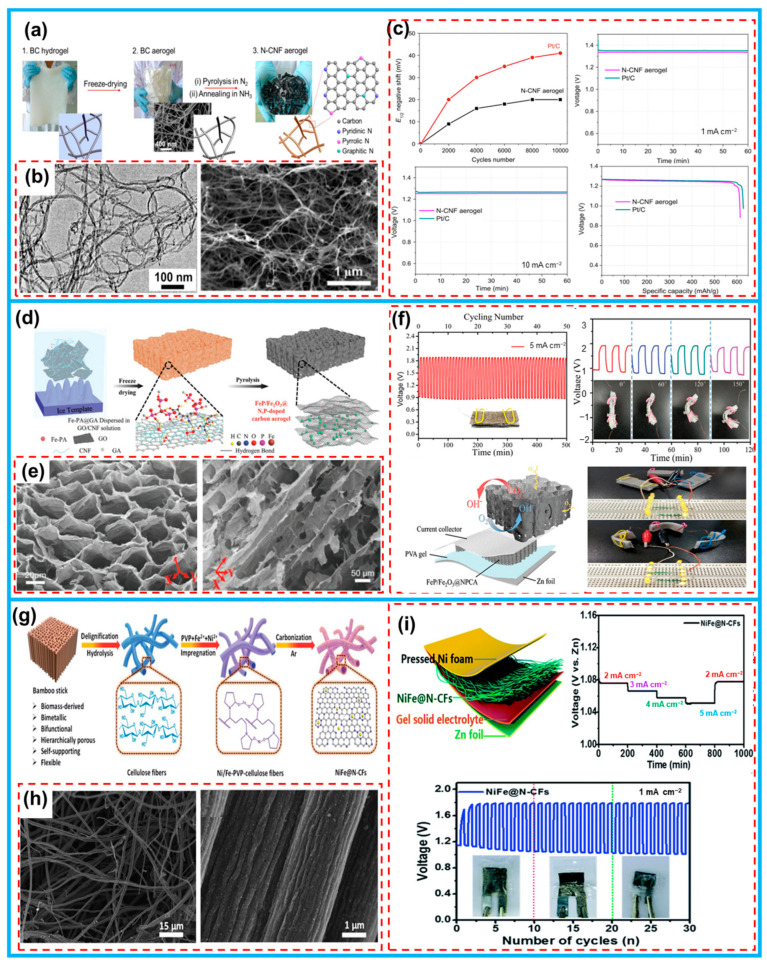
(**a**) Schematic synthesis illustration of N-CNF aerogel. (**b**) SEM pictures of N-CNF aerogels. (**c**) Typical and long-term galvanostatic discharging curves’ performance of N-CNF aerogels and commercial catalysts in Zn–air batteries. (**d**) The preparation process of FeP/Fe_2_O_3_@NPCA. (**e**) SEM pictures of the as-prepared FeP/Fe_2_O_3_@NPCA. (**f**) Cycle galvanostatic charge–discharging and stability of all-solid Zn–air battery with FeP/Fe_2_O_3_@NPCA. (**g**) Synthesis illustration of NiFe@N-CFs. (**h**) SEM images of as-prepared NiFe@N-CFs. (**i**) The flexible quasi-solid-state Zn–air battery with NiFe@N-CFs cathode and galvanostatic curves at different current densities. (From Refs. [113,179,180]).

Yu et al. obtained high-density nitrogen active site-doped carbon nanofibers (N-CNF) through ammonia activation from bacterial cellulose (Figure 15a), and the material inherited the 3D nanofibrous network of biomass with a high specific surface area (Figure 15b) [179]. N-CNF showed better ORR activity and high stability in the alkaline medium, exhibiting high voltages of 1.34 and 1.25 V at discharge currents of 1.0 and 10 mA cm^−1^, respectively, comparable to commercial Pt/C catalysts (Figure 15c). In addition to being used independently, biomass-derived flexible carbon architectures can also serve as a decorated platform for catalysts, leading to the formation of integrated electrodes. Lu et al. employed a strategy combining metal nanoparticles and carbon materials, in which biomass glucosamine was chemically etched on disordered carbon and in situ Fe_3_O_4_ nanoparticles were generated on 3D graphene/nanofibrous layers after etching (Figure 15d) [113]. The foldable aerogel can be directly utilized as a free-standing air electrode for zinc–air batteries without any additives, with a specific capacity of 676 mAh g^−1^ at current densities of 5 mA cm^−2^, energy densities of 517 Wh kg^−1^, and good cycling stability (Figure 15f). Chen et al. proposed to modify the dual-binary metal bifunctional catalysts by doping N element on the basis of a biomass bamboo stick material to obtain the NiFe@N-CFs (Figure 15g). With a fiber network rich in macroporous structure (Figure 15h), the more active sites of the alloy were exposed. The materials were used as self-supported air-positive electrodes in flexible quasi-solid-state ZABs and exhibited high cycling efficiency and mechanical stability (Figure 15i) [180].

Table 1 lists some typical applications of biomass-derived flexible architectures in the field of energy storage, including information on biomass sources, specific surface area, and electrochemical performance. The table reveals that flexible carbon architectures derived from biomass exhibit elevated specific surface areas and excellent electrochemical performance, encompassing remarkable power and energy densities, alongside robust electrochemical stability. These findings further underscore the prospective utility of biomass-derived flexible carbon architectures within the realm of future flexible energy storage devices.

## 4. Conclusions and Perspectives

In the future, electrochemical energy storage technologies are expected to shift from rigid devices towards flexible structures that are stretchable, bendable, and foldable, showcasing advantages, such as enhanced application flexibility, improved durability, and increased spatial utilization. Therefore, it is imperative to accelerate the research efforts concerning novel materials, processing techniques, and flexible energy storage device designs. This comprehensive review summarized the progress of high-performance self-supporting electrode materials derived from renewable resources, covering biomass sources, such as cellulose and lignin; the fabrication methods of binder-free carbonaceous materials, including direct pyrolysis, electrospinning, and vacuum filtration;, and a discussion of freestanding carbon electrodes for energy storage devices. The abundant variety of biomass sources coupled with versatile fabrication methods endowed biomass-derived flexible carbon architectures with diversity, modulability, and customization, enabling their better adaptation to specific energy storage application contexts. The flexible and foldable nature enabled their utilization as self-supporting materials without the need for additional binders or the incorporation of metal current collectors, thus mitigating costs, unnecessary weight, and contact resistance. The distinctive three-dimensional conductive network formed after carbonization offered pathways for charge and ion transfer, while inherent heteroatoms served as pivotal functional groups anchoring active metal species. These unique attributes position biomass-derived flexible carbon architectures as potent drivers of engineering innovation in electrochemical energy storage devices, encompassing supercapacitors, lithium-ion batteries, and zinc–air batteries, among others.

Despite these great achievements, biomass-derived architectures for energy storage devices still face several challenges that need to be overcome to fully harness their application potential:

(1) The conductivity of biomass-derived carbon architectures still exhibits certain differences compared to commercially available carbon papers or carbon cloths. Commercially available carbon papers and carbon cloths typically undergo high-temperature graphitization treatment, resulting in elevated purity and crystallinity that promote the formation of well-carbonized structures and efficient conducting networks. In contrast, the preparation process of biomass-derived carbon architectures may be comparatively simpler, and their conductivity is influenced by the original raw materials, potentially leading to the presence of impurities or defects, resulting in a slight disparity in conductivity compared to commercial carbon papers and carbon cloths. Therefore, novel measures, including interface engineering, nanoscale structure design, and chemical modifications, need to be developed to further enhance their conductivity and unlock their full potential for applications in electrochemical energy storage and related domains.
(2) Further enhancement of the mechanical strength of flexible carbon architectures is necessary. In practical applications, flexible electrodes are subjected to external mechanical forces, such as pressure, tension, or bending. Therefore, they must exhibit excellent mechanical strength to ensure a stable morphology and structure during usage, thereby enhancing the durability and stability of the electrode. At present, certain biomass-derived carbon electrodes fall short of meeting the requirement to maintain their original electrochemical performance, even after extensive folding, necessitating the development of novel approaches to further enhance their mechanical properties. Moreover, future studies should comprehensively analyze the mechanical properties of flexible electrode materials, including tensile strength, foldability, and other relevant aspects, to meet the demands of future flexible devices.
(3) The diverse origins of biomass may lead to variations in the structure, composition, and performance of carbon materials, which can in turn influence the electrochemical performance of electrodes. Therefore, it is essential to conduct a comparative analysis of biomass-derived materials from different regions to assess their respective strengths and weaknesses comprehensively. This approach will provide greater insights into the characteristics of these materials and identify potential areas for improvement, thereby facilitating the optimization and innovation of carbon electrode materials.
(4) Enhancing the surface modulation of biomass-derived carbon architectures for improved electrochemical performance. Due to the abundant natural functional groups present in biomass, which serve as effective carriers for anchoring metal compounds, there is significant attention on utilizing them as favorable platforms. However, the aspect of surface modulation in biomass-derived carbon architectures has received comparatively less focus; the roles played by various functional groups in this context are not yet clearly understood. Consequently, it becomes crucial for future advancements in biomass-derived carbon materials to emphasize surface modulation to further enhance their electrochemical performance and gain a greater understanding of the underlying mechanisms.

## Figures and Tables

**Table 1:** Biomass-derived carbon architectures in energy storage devices.

Biomass	Electrode Material	S_BET_ (m^2^·g^−1^)	V_t_ (cm^3^·g^−1^)	Application	Electrochemical Performance	Ref.
Raw wood	ZIF-67@wood (N:9.34 at%O:5.82 at%Co:1.87 at%)	291.37	0.195	Supercapacitors	EDLC, 101.74 μWh cm^−2^, 5 mW cm^−2^, 10k cycles, 100%	[54]
	Co(OH)_2_/carbonized wood composite (Co(OH)_2_: 5.7 mg cm^−2^)	568.13			EDLC, 0.69 mWh cm^−2^, 1.126 W cm^−2^, 10k cycles, 85%	[65]
	N-doped porous carbon monoliths (N: 3.2wt%)	708.2	0.38		HSC, 9.3 Wh m^−2^, 248.3 W m^−2^, 5k cycles, 94%	[66]
	N-doped carbon aerogels	2183	1.83		EDLC, 5.5 W h kg^−1^, 3.7 kW kg^−1^, 10k cycles, 93.6%	[68]
Fabric	N-doped activated carbon cloth	2116	1.458		EDLC, 215.9 F g^−1^ (1 A g^−1^), 98%, 20k cycles, 98%	[75]
	N-doped carbon interface				EDLC, 3625 mF cm^−2^, 1.06 mWh cm^−2^, 10k cycles, 96%	[82]
Silk	Carbonized silk fabric-MnO_2_	25.85			EDLC, 14.58 W h kg^−1^, 0.25 kW kg^−1^, 10k cycles, 100%	[89]
Paper	B, N, and O heteroatom-doped 3D interconnected carbon microfiber networks	1382.5	0.973		EDLC, 12.4 W h kg^−1^, 300.6 kW kg^−1^, 242.4 F g^−1^, 100 cycles, 100%	[23]
	Cellulose-based activated carbon fiber papers	808	0.52		PC, 48.8 F cm^−3^, 134.1 F g^−1^, 10k cycles, 100%	[92]
	Activated carbon fibers	762.65	0.356		EDLC, 56.25 mWh cm^−2^, 997 mW cm^−2^, 5k cycles, 100%	[34]
Cotton stalks	Integrated paper electrodes	1972	0.369		HSC, 331 μW h cm^−2^, 0.3 mW h cm^−3^, 10k cycles, 97.1%	[116]
Cellulose	Cellulose–polypyrrole@reduced graphene oxide composite electrodes	24.8			EDLC, 489 mF cm^−2^ (0.5 mA cm^−2^), 1k cycles, 100%	[25]
Carboxymethylcellulose	MXene/cellulose/carbon nanotube composite electrodes	95.2			HSC, 258.8 μWh cm^−2^, 750 μW cm^−2^, 15k cycles, 93.2%	[118]
Bamboo pulp	Super-flexible porous carbon fibrous film	309	0.178		EDLC, 10.3 W h kg^−1^, 250 W kg^−1^, 8k cycles, 94.6%	[121]
Bacterial cellulose	Carbon aerogels	893	0.30		EDLC, 297 F g^−1^ (1 A g^−1^), 14.83 Wh kg^−1^ (0.60 kW kg^−1^), 65k cycles, 100%	[38]
Raw cotton	Porous carbon fibers	2124.9	1.01	Lithium batteries	778 mA h g^−1^ (0.2 C), 450 mA h g^−1^ (0.5 C), 300 cycles, 99%	[73]
Silk	Carbonized silk @Si@graphene				1070 mAh g^−1^ (200 mA g^−1^), 300 cycles, 99%	[87]
	N/O-co-doped carbonized fibroin (N: 3.4 at% O: 7.9%)	20.8			5.6 mAh cm^−2^, 457.2 Wh L^−1^, 300 cycles, 99.8%	[85]
Bacterial cellulose	BC aerogels	375			1134 mA h^−1^, 700 mA h^−1^ (400 mA g^−1^), 400 cycles, 98.3%	[102]
	O*_v_*-ZnO@CBC aerogels	143			710 mAh g^−1^ (1 A g^−1^), 1k cycles, 99%	[103]
Cellulose nanofibril	FeP/Fe_2_O_3_@N,P-doped carbon aerogel (N:3.45at%, P:8.12at% Fe:0.84at%)	503.43		Zn–air batteries	676 mAh g^−1^, 517 Wh kg^−1^ (5 mA cm^−2^), 100 h, 46.17%	[113]
Raw wood	N-doped hierarchical porous carbon (N: 3.7 at%)	1039			801 mA h g^−1^, 955 W h kg^−1^, 110 h, 58%	[64]
Dried miscanthus stems	Inner@Co@CNTs				175.5 mW cm^−2^, 0.27 V (5 mA cm^−2^), 24 h, 81%	[181]
Raw wood	FeCo@NS-CA aerogels				140 mW cm^−2^, 760 mA h g^−1^, 400 h	[182]
	Co/CoO@NWC				800 mAh g^−1^, 0.84 V, 270 h	[183]
Lignin	BNF-LCFs				791.5 mAh g^−1^, 1.536 V, 200 h, 54.6%	[126]

## Data Availability

Not applicable.
